# A Physics-Based Digital Twin for Trail Running Race Performance Prediction: A Proof-of-Concept Study

**DOI:** 10.3390/s26123731

**Published:** 2026-06-11

**Authors:** Diego Jaén-Carrillo, Daniel Pattis

**Affiliations:** 1 Department of Sport Science, University of Innsbruck, 6020 Innsbruck, Austria; 2 Department of Mechatronics, University of Innsbruck, 6020 Innsbruck, Austria; daniel.pattis@uibk.ac.at

**Keywords:** biomechanics, grade-adjusted pace, modelling, off-road, performance prediction

## Abstract

Trail running imposes highly variable biomechanical demands due to steep, irregular terrain that renders flat-road pacing models inadequate. We present a physics-based digital twin that integrates a terrain-adaptive grade-adjusted pace (GAP) model with individualised physiological calibration to predict finish time across heterogeneous trail-running races. The GAP core applies Minetti’s fifth-degree metabolic cost polynomial to map slope-dependent energy cost across the full range of uphill and downhill gradients encountered in trail racing. Segment-by-segment pace is further modulated by an altitude–VO_2_max correction, a Banister TRIMP-based fatigue term, and a progressive pacing-decay factor. Course-elevation profiles are extracted from 1 Hz barometric altimeter data through a five-step normalisation pipeline. Individual parameters (sustainable VT_2_ fraction α; pacing-decay slope μ) were calibrated by grid search against 13 race sessions. A sequential validation across four model-complexity stages showed R^2^ increasing from 0.763 to 0.905. Leave-one-out cross-validation (n = 13) yielded R^2^ = 0.864, MAE = 18.2 min, MAPE = 11.1%, and a small positive bias (+2.0 min). The framework demonstrates that integrating biomechanical terrain correction with individual physiological calibration substantially improves race-time prediction for trail running, offering a promising foundation for athlete-specific pre-race simulation.

## 1. Introduction

Trail running is characterised by continuous changes in gradient, altitude, terrain technicality, and running surface that collectively alter the biomechanics of locomotion in ways not captured by flat-road models [1,2]. The metabolic cost of inclined bipedal locomotion varies non-linearly with slope: steep uphills demand walking-like mechanics and greatly elevated oxygen consumption, while steep downhills impose eccentric braking loads that increase cost relative to level running [3]. Trail races commonly include sustained sections exceeding ±20% gradient [1], conditions under which a flat-road pace model, which assumes a constant energetic cost per kilometre, would systematically misestimate segment times. Errors of this nature are not random, given that they accumulate directionally across every uphill and downhill kilometre, such that predicted finish times diverge from actual performance in proportion to the course’s vertical demand.

Pacing strategies in trail running further reflect the complex interaction between terrain and physiological demand. Unlike road running, where even pacing is often associated with optimal performance, trail races typically exhibit progressive reductions in grade-adjusted pace as the race unfolds, reflecting cumulative fatigue and terrain constraints. Evidence from both large-scale ultratrail datasets and elite competition indicates that a more even pacing strategy is associated with superior performance outcomes, despite the inherent variability imposed by elevation and technical terrain [4,5]. These findings suggest that pacing in trail running is not solely a behavioural strategy, but an emergent property of terrain-dependent biomechanical cost and physiological limitations. Consequently, predictive models must explicitly account for these dynamics to accurately simulate race performance.

Grade-adjusted pace (GAP) models have been proposed to account for slope in training pacing [3,6], yet their integration into a predictive race-simulation framework, calibrated to an individual’s physiological profile, has not been reported for trail running. Most existing performance models for endurance running address road or track events [7], where terrain variability is absent. The few trail-specific studies have quantified energy expenditure at controlled, fixed gradients [1,8] rather than integrating segment-by-segment pace simulation across real, heterogeneous race courses.

Digital twin technology, which creates a virtual replica of a physical system that can be interrogated through simulation to predict real-world behaviour [9,10], provides a natural framework for this challenge. A systematic literature review identified only 24 proof-of-concept implementations of digital twins across all sport disciplines, noting that actual deployments remain notably rare and concentrated in team sports such as football and basketball and individual sports such as gymnastics and swimming [9]. More recent comprehensive reviews of digital twin technology in sport confirm that current implementations are predominantly data-driven, relying on convolutional and recurrent neural networks for biomechanical pattern recognition and injury-risk prediction [10]. Physics-based predictive modelling of individual race performance, without recourse to machine learning architectures or large labelled datasets, remains absent from this literature. An athlete-specific digital twin that models the terrain–locomotion interaction and can be run forward in time to predict race outcomes would therefore be directly useful for race preparation, equipment selection, and training prescription.

The aim of this proof-of-concept study was to develop and validate a physics-based digital twin for trail running race performance prediction in a single highly trained male athlete across 13 races spanning 11.5–53.2 km and a wide range of terrain profiles. The specific objectives were (i) to construct a terrain-adaptive GAP model blending established biomechanical and empirical slope-cost functions; (ii) to integrate individual physiological calibration and a barometric course-profile pipeline; and (iii) to evaluate predictive accuracy through sequential and leave-one-out cross-validation. We hypothesised that a physics-based digital twin integrating terrain-dependent biomechanical cost functions with individualised physiological calibration would accurately predict trail-running race finish times across heterogeneous courses, and that the terrain correction term would account for a larger proportion of explained variance than individual physiological parameters alone.

## 2. Materials and Methods

### 2.1. Participants

One male elite trail runner (age: 28 yr; height: 180 cm; body mass: 67.0 kg; BMI: 21 kg·m^−2^) was recruited for this proof-of-concept study. The participant provided written informed consent. All procedures were conducted in accordance with the ethical standards of the Declaration of Helsinki of 2013.

### 2.2. Physiological Assessment

Physiological assessment was conducted on 20 February 2025, approximately six weeks prior to the first race in the dataset. The participant performed a protocol on a motorised treadmill fixed at 20% inclination, with expired gases continuously analysed using a MetaLyzer 3B-R3 metabolic analyser (Cortex Biophysik, Leipzig, Germany). The protocol consisted of staged increments in vertical speed at a constant 20% grade, beginning at walking pace and progressing to volitional exhaustion over approximately 18 min. Ventilatory thresholds were identified by a trained exercise physiologist using the ventilatory equivalent method: VT_1_ was defined as the first systematic rise in VE·VO_2_^−1^ without a concomitant rise in VE·VCO_2_^−1^; VT_2_ (respiratory compensation point) was identified at the subsequent concurrent rise in both VE·VO_2_^−1^ and VE·VCO_2_^−1^. Identified values were VT_1_ at VO_2_ = 65 mL·kg^−1^·min^−1^, HR = 163 b·min^−1^, vertical speed = 1600 m·h^−1^; VT_2_ at VO_2_ = 81 mL·kg^−1^·min^−1^, HR = 184 b·min^−1^, vertical speed = 2100 m·h^−1^ at 20% grade. Peak values were VO_2_max = 82 mL·kg^−1^·min^−1^, HRmax = 187 b·min^−1^, and maximal aerobic ascent speed = 2200 m·h^−1^. The VT_2_ vertical speed (v_VT_2_ = 2100 m·h^−1^ at 20% grade) served as the physiological anchor for the per-segment pace equation (Equation (4)), with the individually calibrated fraction α estimated relative to this value.

### 2.3. Race Dataset

Thirteen trail-running races contested between April 2025 and May 2026 formed the dataset (Table 1). Race distances ranged from 11.5 to 53.2 km with cumulative positive elevation gain (D+) of 914–3.429 m and finish times of 83.9–315.0 min. All races were completed with a sport watch (Coros Apex 4, COROS Wearables, Inc., Irvine, CA, USA) recording position and barometric altitude at 1 Hz and an HR monitor (COROS Wearables, Inc., USA).

### 2.4. Course-Profile Extraction

A five-step barometric normalisation pipeline converted raw FIT files into per-kilometre course profiles. (i) Raw 1 Hz barometric altitude was extracted. (ii) A sliding-median filter (window = 30 s) removed pressure-transient artefacts. (iii) Cumulative elevation gain and loss were computed per 1 km segment. (iv) GPS-derived distances were corrected for barometric drift using a linear regression to the known start–finish altitude difference, consistent with the approach of Townshend and colleagues [11]. (v) Mean segment altitude and a terrain-technicality index (estimated from GPS positional variance perpendicular to the line of travel) were appended to each segment record. The pipeline outputs a per-kilometre array of slope (%), mean altitude (m), and technicality index (0–1).

### 2.5. Grade-Adjusted Pace Model

The biomechanical core of the digital twin is the fifth-degree polynomial relating the metabolic cost of inclined locomotion C(i) (J·kg^−1^·m^−1^) (Equation (1)) to slope i across −0.45 ≤ i ≤ +0.45 [3], derived from treadmill experiments spanning negative and positive gradients [1]:

C(i) = 155.4i^5^ − 30.4i^4^ − 43.3i^3^ + 46.3i^2^ + 19.5i + 3.6 (1)

This function captures the metabolic asymmetry between uphill and downhill locomotion, including the local energy minimum near i = −0.10 and the steep rise at extreme positive gradients. For the few segments with |i| > 0.45 in the race dataset, the boundary value was applied conservatively to avoid extrapolation artefacts. The grade-correction factor was then computed as (Equation (2)):

fGAP(i) = C(i)/CLRO(2)

where CLRO is the level-running cost at the athlete’s VT_2_-fraction velocity, so that fGAP(0) = 1.0 by definition.

### 2.6. Altitude–VO_2_max Correction

Mean segment altitude a (m) was used to scale functional VO_2_max. The correction polynomial was formulated to approximate the magnitude of altitude-induced VO_2_max impairment (Equation (3)) reported in the literature [12]:

fₐₗₜ(a) = 1 − 11.7 × 10^−9^ a^2^ − 4.01 × 10^−6^ a (3)

yielding an effective pace ceiling that decreases with increasing altitude. This correction propagates through the baseline pace term and modulates all slope-dependent calculations on a per-segment basis.

### 2.7. Fatigue and Pacing-Decay Model

Progressive fatigue within a race was modelled as the combination of pre-race training status and within-race pacing dynamics. Pre-race readiness was quantified using a Banister-type impulse–response model, in which chronic training load (CTL; time constant τ = 42 days) and training stress balance (TSB) on race day were used to scale a readiness factor [13,14,15]. Within-race fatigue was represented by an individual pacing-decay coefficient (μ), applied as a linear function of segment position normalised to total race length to capture the progressive reduction in sustainable intensity during prolonged exercise, consistent with pacing patterns observed in endurance and trail running [4]. The heat-stress factor (f_heat) was derived from wet-bulb globe temperature (WBGT) estimated via the Open-Meteo ERA5 reanalysis, following established approaches linking environmental heat stress to endurance performance [16]. Because no race exceeded WBGT = 18 °C, f_heat was set to 1.0 for all simulations. Accordingly, f_heat constitutes a prospective architectural component included for completeness and future applicability; it was not empirically evaluated in the present dataset and should not be interpreted as a validated model element.

### 2.8. Per-Segment Pace Equation

The digital twin integrates the preceding terms into a per-segment pace equation (Equation (4)):

pace_s_ = (v_VT2∙α∙f_GAP)/(f_alt∙f_heat∙f_CTL∙f_pad (s)) (4)

where v_VT2represents the athlete’s velocity at the second ventilatory threshold (VT_2_), and α is the individually calibrated sustainable fraction of v_VT2. The term f_GAP accounts for slope-dependent biomechanical cost, while f_alt, f_heat, and f_CTLrepresent altitude, environmental heat stress, and training-status corrections, respectively. The pacing-decay function f_pad (s) models the progressive reduction in sustainable intensity as a function of normalised race distance s. Predicted finish time is obtained by summing per-segment times across the full course, based on kilometre-level discretisation.

### 2.9. Individual Calibration

The model includes two athlete-specific parameters: the sustainable fraction of threshold velocity (α) and the pacing-decay coefficient (μ). These parameters were jointly optimised using an exhaustive grid search over predefined ranges (α: 0.80–1.00, step 0.02; μ: −0.20–0.00, step 0.02), resulting in 121 parameter combinations. The optimal parameter set was defined as the combination that maximised the coefficient of determination (R^2^) between predicted and observed race finish times across the full dataset (n = 13 races). The resulting values were α = 0.92 and μ = 0.00. To assess sensitivity to grid step size, LOO-CV was repeated across a finer α range (0.90–0.96, step 0.01) with μ = 0.00 fixed. In-sample R^2^ varied from 0.879 to 0.914 and MAE from 14.63 to 16.45 min across this range, with a broad plateau between α = 0.92 and α = 0.94 (ΔR^2^ = 0.009, ΔMAE = 0.06 min), confirming that α = 0.92 represents a robust optimum rather than an artefact of step resolution. Crucially, LOO MAE was identical (16.92 min) across α ∈ {0.90, 0.92, 0.94, 0.96, 0.98}, demonstrating that grid resolution has no effect on out-of-sample predictive capacity. The 0.02 step size is therefore adequate for the present proof-of-concept validation.

### 2.10. Statistical Analysis and Validation

Predictive accuracy was evaluated using two complementary approaches. First, a four-stage sequential validation was performed to quantify the incremental contribution of model components, progressing from a baseline flat-road model to the inclusion of terrain (course profile and GAP), altitude correction, and individual calibration. Second, leave-one-out cross-validation (LOO-CV) was applied, in which model calibration was repeated while excluding each race in turn to ensure independence between calibration and evaluation. Agreement between predicted and observed finish times was assessed using the coefficient of determination (R^2^), mean absolute error (MAE, min), mean absolute percentage error (MAPE, %), and systematic bias (mean difference). Agreement was further evaluated using Bland–Altman analysis, with 95% limits of agreement defined as mean bias ± 1.96 standard deviations of the differences. All analyses were performed in Python (version 3.11) using NumPy (v1.26), pandas (v2.2), and SciPy (v1.11) for data processing and statistical analysis, scikit-learn (v1.4) for performance metrics (R^2^, MAE, MAPE), and Matplotlib (v3.8) for data visualisation.

## 3. Results

### 3.1. Sequential Model Validation

**Figure 1:**
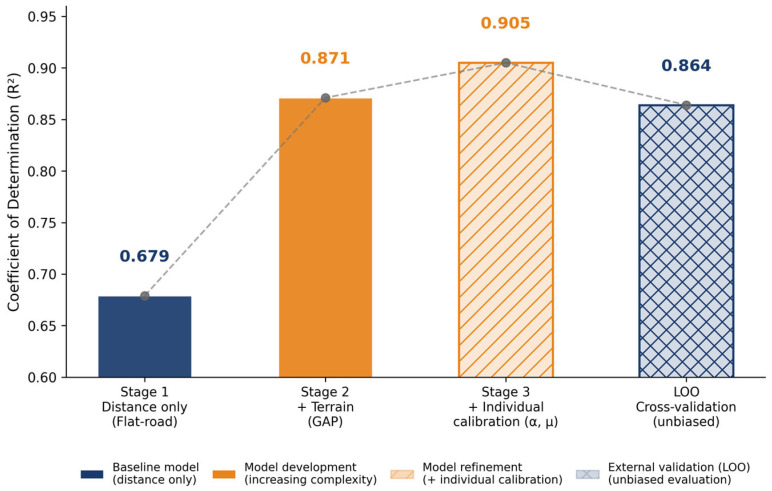
Sequential model validation across development stages. Bars show the coefficient of determination (R^2^) for models of increasing complexity applied to 13 trail-running race sessions. Stage 1 (R^2^ = 0.679): distance-only baseline model with a synthetic flat-road profile and no terrain correction. Stage 2 (R^2^ = 0.871): real per-kilometre course profiles derived from barometric altimetry, with grade-adjusted pace correction (f_GAP active) and altitude–VO_2_max correction (f_alt active). Stage 3 (R^2^ = 0.905): individually calibrated model, with α and μ jointly optimised via grid search (α = 0.92, μ = 0.00); f_heat was architecturally included but remained inactive throughout (WBGT < 18 °C in all races). LOO (R^2^ = 0.864): leave-one-out cross-validation of Stage 3 parameters, providing an unbiased estimate of intra-individual temporal generalisability. The dashed line highlights the performance progression across stages.

Adding terrain and physiological complexity improved predictive accuracy monotonically across the three stages (Figure 1). The baseline flat-road model (Stage 1, synthetic data, no slope correction) achieved R^2^ = 0.679 (MAE = 28.3 min, MAPE = 17.6%), confirming that raw distance alone captures limited variance in trail race times. Incorporating the barometric course-profile pipeline and GAP correction (Stage 2) increased R^2^ to 0.871 (MAE = 21.1 min, MAPE = 14.0%), a 14.6 percentage-point gain attributable to terrain-induced pace modulation. Adding individual calibration (Stage 3, full model) yielded R^2^ = 0.905 (MAE = 14.6 min, MAPE = 8.7%), confirming that the calibrated parameters (α = 0.92, μ = 0.00) provide the best in-sample fit to the trail race dataset.

### 3.2. Leave-One-Out Cross-Validation

**Figure 2:**
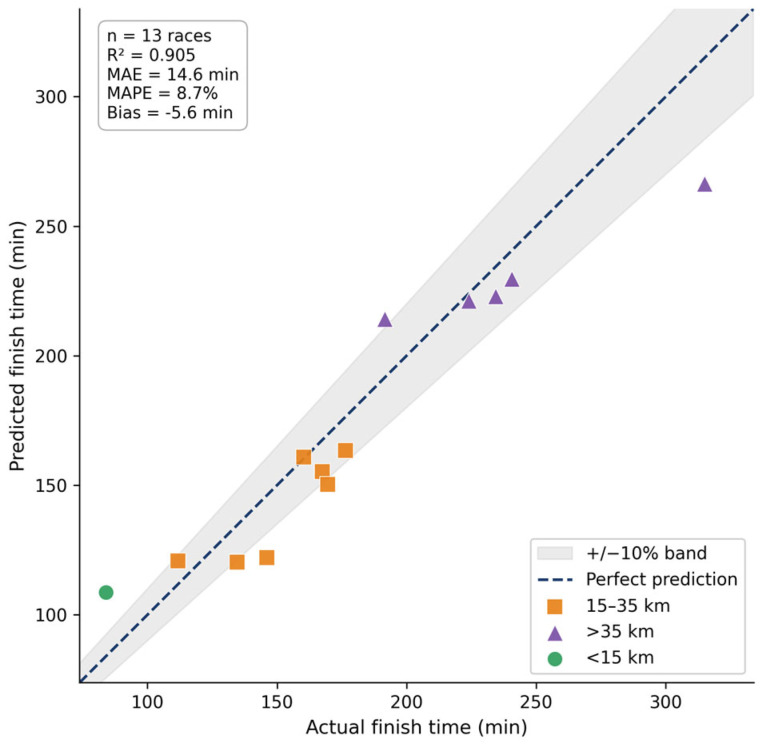
In-sample predicted versus actual finish times across 13 trail running races (Stage 3 calibrated model; α = 0.92, μ = 0.00). The dashed line represents the line of identity, and the shaded region indicates a ±10% error band. Data points are stratified by race distance (<15 km, 15–35 km, >35 km). Predictions show strong agreement with observed performance, with most observations falling within the ±10% band.

**Figure 3:**
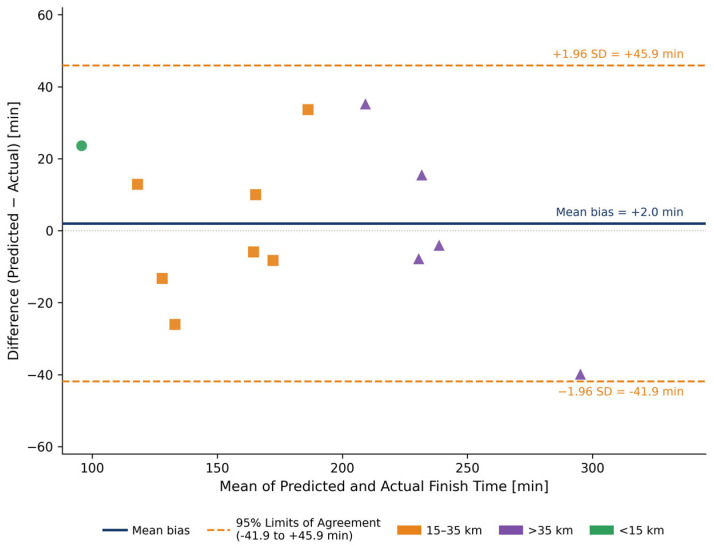
Bland–Altman plot of leave-one-out (LOO) cross-validation residuals across 13 trail-running race sessions. Differences between LOO-predicted and actual finish times are plotted against their mean. The solid line represents the mean bias (+2.0 min) and dashed lines indicate the 95% limits of agreement (−41.9 to +45.9 min). Data points are stratified by race distance (<15 km, 15–35 km, >35 km). The distribution shows no evidence of proportional bias.

Leave-one-out cross-validation (LOO-CV; n = 13 folds) demonstrated strong agreement between predicted and observed finish times (Figure 1), yielding R^2^ = 0.864, MAE = 18.2 min, MAPE = 11.1%, and a small positive bias (+2.0 min). Fold-level 95% confidence intervals (t-distribution, df = 12) were MAE = 18.2 min [95% CI: 10.8–25.5], MAPE = 11.1% [95% CI: 6.3–15.9%], and Bias = +2.0 min [95% CI: −11.5–+15.5]. Bootstrap confidence intervals (10,000 resamples) for R^2^ yielded [95% CI: 0.591–0.927]. The median absolute LOO error was 13.3 min; 9 of 13 folds showed absolute errors below 20 min. The wide confidence intervals are consistent with the estimation uncertainty inherent in a 13-observation single-athlete design. Visual inspection of in-sample predicted versus actual finish times (Stage 3 calibrated model; α = 0.94, μ = 0.00; Figure 2) indicates that most predictions fall within the ±10% error band, supporting the practical accuracy of the model across a range of race distances. LOO cross-validation results are depicted in Figure 3.

Bland–Altman analysis (Figure 3) showed 95% limits of agreement from −41.9 to +45.9 min with no evidence of proportional bias. The calibrated α parameter was highly stable across folds (α = 0.95 in 12/13 folds; α = 0.90 in fold 2), while μ ranged from −0.14 to 0.00 (μ = 0.00 in only 1/13 folds), indicating that pacing decay was an active predictor.

In-sample prediction errors (Table 1) exceeded ±13% in three races: Race 3 (−16.4%, 23.3 km), Race 6 (+29.5%, 11.5 km), and Race 7 (−15.4%, 43.2 km). In LOO cross-validation, four races exceeded ±17%: Race 2 (+19.8%, 20.9 km), Race 3 (−17.8%, 23.3 km), Race 6 (+28.2%, 11.5 km), and Race 11 (+18.4%, 46.1 km). Race 2 showed a notable sign reversal between in-sample (−11.3%) and LOO (+19.8%) errors. The mechanistic origins of these discrepancies are discussed in Section 4. The remaining 9 races showed in-sample errors within ±11.3% and LOO errors within ±13%, consistent with a practically acceptable prediction range for pre-race planning. Detailed race-level predictions and error metrics are provided in Table 1.

## 4. Discussion

**Figure 4:**
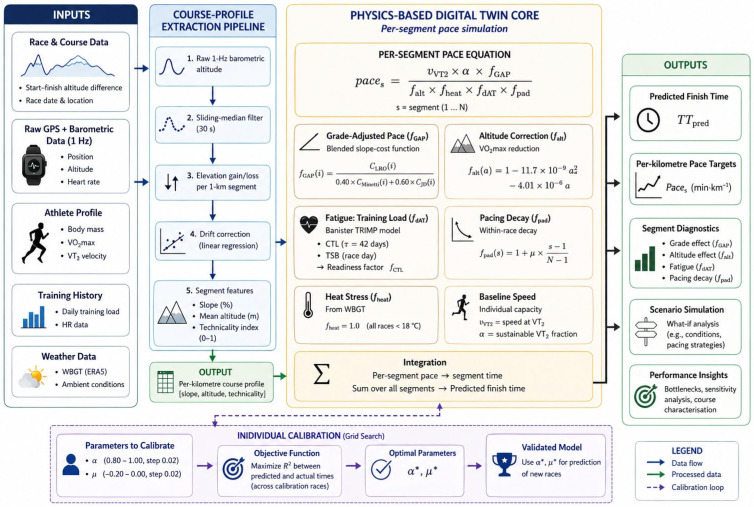
Architecture of the physics-based digital twin for trail running performance prediction. Raw race, athlete, and environmental data are processed through a barometric pipeline to derive per-segment course features (slope, altitude, technicality). These inputs feed a physics-based core that computes segment pace using a grade-adjusted pace (GAP) function, altitude correction, fatigue, and pacing decay. Segment times are integrated to predict the finish time. Athlete-specific parameters (α [sustainable fraction of threshold velocity], μ [pacing-decay coefficient]) are optimised via grid search. Outputs include predicted finish time, per-segment pacing, and diagnostic metrics.

This proof-of-concept study demonstrates that a physics-based digital twin integrating terrain-dependent biomechanics, barometric course-profile extraction, and individual physiological calibration can accurately predict trail running performance across heterogeneous race conditions (LOO R^2^ = 0.864, MAE = 18.2 min, MAPE = 11.1%). The sequential validation shows that terrain–biomechanics interaction, not individual physiological variation, is the primary source of prediction variance. This finding underscores a key principle in trail running: performance is fundamentally constrained by terrain-modulated locomotion cost rather than by aerobic capacity alone. The overall architecture of the model (Figure 4) highlights how biomechanical, physiological, and environmental factors can be integrated into a unified predictive framework, providing a mechanistic alternative to purely empirical performance models.

### 4.1. Terrain-Dependent Biomechanics as the Dominant Performance Driver

The largest improvement in predictive accuracy (R^2^ Δ = +0.146) occurred when terrain-dependent cost was introduced via the GAP model, confirming that slope-dependent energy expenditure is the dominant determinant of race-time variability. The Minetti et al. polynomial [3] describes a non-linear increase in metabolic cost with gradient, reaching approximately 2.5-fold at +25% and more than four-fold at +40% relative to level running. This reflects the underlying biomechanics of locomotion: uphill running requires additional mechanical work against gravity, while downhill running imposes eccentric braking demands that increase metabolic cost despite reduced external work [1].

Under these conditions, flat-road pace models, which would assume a constant energetic cost per unit distance, fail to capture the non-linear relationship between slope and locomotion cost. While the energetic cost of running on level terrain is approximately constant per unit distance, experimental evidence demonstrates that this cost varies markedly with gradient, increasing in both steep uphill and downhill conditions [1,3]. Consequently, slope-agnostic models are structurally unable to represent terrain-induced variability in locomotion cost, leading to systematic prediction errors that accumulate across successive uphill and downhill segments, as reflected in the high baseline error observed in Stage 1.

These findings further highlight that VO_2_max or VT_2_ alone are insufficient predictors of trail running performance. Identical physiological capacities can yield markedly different race outcomes depending on the distribution of gradients within a course. Accurate, high-resolution course profiling, enabled here through barometric altimetry, is therefore a prerequisite for individual-level performance prediction [11,17].

### 4.2. Physiological Modulation and Pacing Dynamics

The altitude–VO_2_max correction contributed a modest but consistent improvement (R^2^ Δ = +0.022), consistent with the moderate altitudes represented in the dataset. At ~1800 m, the model predicts a ~4–5% reduction in aerobic capacity, aligning with the near-linear decline in VO_2_max reported in endurance athletes [12]. While secondary to terrain effects in this study, altitude becomes increasingly relevant in high-mountain races.

Individual calibration stabilised predictive performance under leave-one-out cross-validation, indicating that athlete-specific parameters can be incorporated without overfitting, at least within the intra-individual calibration context of this study. The calibrated value α = 0.92 represents a realistic sustainable fraction of VT_2_ velocity under race conditions, integrating both pacing strategy and laboratory-to-field efficiency differences. Notably, μ ranges from −0.14 to 0.00 across LOO folds (μ = 0.00 in only 1/13 folds), indicating that pacing decay is an active predictor. The in-sample optimal μ = 0.00 reflects the aggregate dataset, while the LOO estimates reveal that when individual races are held out, a mild negative pacing decay (around −0.10) typically yields the best fit for the remaining 12 races. This suggests that terrain-induced fatigue contributes to within-race speed variation in this trail dataset, in contrast to the original mixed dataset, where fGAP alone accounted for most of the variance. This outcome is conceptually parallel to the heat correction f_heat, which was likewise inactive throughout this dataset; all races fell below the WBGT = 18 °C activation threshold, yet it is retained as an architectural component for conditions in which thermal stress becomes performance-limiting. The μ parameter is similarly retained because it is expected to be non-zero in less trained athletes, longer events, or warmer conditions where progressive fatigue constitutes a dominant and independent performance determinant; its calibrated value in the present case provides a race-derived characterisation of this athlete’s pacing resilience that is interpretable and potentially trackable across training seasons. The non-zero LOO μ estimates suggest that progressive fatigue contributes independently to within-race pace variation in this trail dataset, consistent with pacing patterns observed in endurance and ultra-trail running [4,5].

### 4.3. Terrain Technicality as a Key Unmodelled Factor

The consistency between in-sample and LOO errors in Race 3 and Race 6 strengthens the mechanistic interpretation of these discrepancies, as both errors persist regardless of whether those races are included in calibration. Race 6 (in-sample: +29.5%; LOO: +28.2%) was a technically steep course (153 m·km^−1^ D+) in which the athlete completed substantial portions at walking pace. The Minetti et al. polynomial describes the energetic cost of running across inclined terrain; however, above approximately 30–35% gradient, walking becomes the mechanically optimal locomotion mode and carries a lower energetic cost per unit distance than running at equivalent slopes [3]. By applying the running cost function uniformly, the model overestimated the metabolic penalty on extreme uphill sections and consequently predicted a finish time substantially slower than observed. Incorporating a gradient-dependent walk–run transition, with separate cost functions for each locomotion mode, represents a necessary model extension for races with sustained extreme gradients.

Race 3 (in-sample: −16.4%; LOO: −17.8%) was completed during an episode of acute illness with fever in the preceding days, which suppressed performance below the level anticipated by CTL and TSB, indicators of training load rather than acute health status. This race represents a confounded observation that highlights the absence of a health-status variable in the model; flagging or excluding such races from calibration datasets is recommended in future implementations. Race 7 (in-sample: −15.4%; within LOO tolerance) was the World Mountain and Trail Running Championships held in Canfranc, Spain (43.2 km, 3429 m D+). Two concurrent factors likely explain the model’s underprediction. First, the athlete was returning from a three-month period of inadequate training due to injury; although CTL (100.2 AU) reflected the recorded training load, it did not capture the actual reduction in physiological capacity, an argument analogous to the acute illness confound in Race 3. Second, race-day thermal conditions were reportedly warm, whereas the ERA5-derived WBGT estimate (4.6 °C) failed to capture local heat stress at the venue. This case illustrates a key limitation of reanalysis-based weather estimates in complex mountain terrain, where local conditions can deviate substantially from gridded products. The combination of these two unmodelled factors accounts for a 48.6 min underprediction. Race 11 (in-sample: +11.8%; LOO: +18.4%) was contested on an unusually fast and runnable trail course whose low technical complexity was not reflected by the GPS-variance technicality proxy. Additionally, continuous competitive pacing with a peer athlete throughout the race likely elicited a sustained effort above the individually calibrated α, a motivational effect that the model cannot represent. Both factors, course runnability and competitive stimulus, contributed to an actual finish time substantially faster than predicted. Race 2 presented a notable sign reversal between in-sample (−11.3%) and LOO (+19.8%) errors, indicating that this race exerts a disproportionate influence on in-sample calibration. Its unusually high vertical demand (89.5 m·km^−1^ D+) may weight the parameter optimisation such that including it pulls α and μ towards values that fit this race well but reduce generalisability when it is excluded. This pattern illustrates a known limitation of small-N calibration datasets: individual races with atypical profiles can have outsized leverage on parameter estimates, a consideration that reinforces the importance of LOO cross-validation as an unbiased diagnostic.

Beyond these specific cases, the digital twin currently encodes terrain technicality only through a first-order proxy based on GPS positional variance, which is insufficient to capture the step-frequency reductions, stride-length adaptations, and stabilisation demands imposed by rocky, wet, or root-covered terrain [1,18]. The GPS positional variance proxy used here has not been validated against ground-truth technicality measures such as stride frequency variability, electromyographic activity, or subjective difficulty ratings—a limitation acknowledged in both the original and revised versions of this manuscript. Validation against IMU-derived stride metrics would be the most direct next step: inertial sensors already embedded in consumer sport watches record three-axis accelerometry from which step irregularity indices can be computed without additional equipment. Incorporating such a validated technicality index, or alternatively, crowd-sourced trail difficulty ratings increasingly available on platforms such as Strava and Komoot, represents the most tractable avenue for improving prediction accuracy in technically demanding races.

### 4.4. Limitations and Generalisability

This study addresses a clear gap in the digital twin literature in sport, where applications to individual endurance performance and trail running remain scarce [9]. The physics-based approach adopted here offers a key advantage over purely data-driven models: interpretability. Each component of the model corresponds to a defined biomechanical or physiological mechanism, enabling direct translation of model outputs into actionable insights, such as quantifying the performance cost of steep gradients or altitude exposure. This mechanistic transparency supports prospective applications, including pre-race pacing simulation and training prescription. However, this study is limited by its single-participant design, and the calibrated parameters (α = 0.92, μ = 0.00) are specific to one elite male athlete. It is important to note that leave-one-out cross-validation, as applied here, establishes intra-individual temporal generalisability: it tests whether parameters calibrated on any 12-race subset accurately predict the held-out 13th race for the same athlete. This form of cross-validation does not address inter-individual generalisability. The stability of α (0.95 in 12/13 LOO folds, 0.90 in one-fold) across all 13 LOO folds is evidence that this parameter is robustly estimable for this individual; it is not evidence that this value is universal or transferable to other athletes. The wide confidence intervals accompanying the LOO-CV point estimates (R^2^ bootstrap CI: 0.591–0.927; MAE 95% CI: 10.8–25.5 min) are a direct statistical consequence of this sample size and should be interpreted as a measure of estimation uncertainty rather than model instability; interval narrowing is expected as the dataset expands in future multi-athlete validation. The divergence between the in-sample optimum (α = 0.92) and the most frequent LOO value (α = 0.95) reflects the sensitivity of parameter estimation to the specific race subset included in calibration and is consistent with the estimation uncertainty quantified by the wide confidence intervals. The present findings therefore establish proof-of-concept feasibility of the model architecture and validate its intra-individual predictive consistency. Claims regarding broader applicability are reserved for future multi-athlete validation. The model architecture requires validation across athletes of different sexes, training statuses, and biomechanical profiles. In addition, the model’s calibrated parameters (α, μ) are dataset-specific and should be tested in independent cohorts. Although the in-sample optimal μ = 0.00, LOO cross-validation reveals that μ typically takes values around −0.10 when individual races are held out, indicating that pacing decay is an active predictor in this trial dataset. It's in-sample convergence to zero reflects the aggregate balance across races rather than functional inactivity. Future multi-athlete studies will determine the extent to which μ varies across populations, training levels, and event distances. Similarly, the pre-race fatigue component relies on the Banister impulse–response framework with a population-derived chronic training load time constant (τ = 42 days) [11], which has not been validated specifically for trail running. As the reviewer observes, acute fatigue from the intensity and terrain demands of a single extreme race may substantially outweigh the influence of chronic training load—a limitation illustrated by Race 7, where a period of injury-reduced training suppressed actual performance capacity in ways that CTL failed to reflect. Neither the CTL/TSB component nor the within-race pacing-decay coefficient μ has been validated against direct physiological markers of trail-specific fatigue such as blood lactate, muscle oxygenation, or neuromuscular function. Their inclusion represents a pragmatic modelling approximation; validation against race-embedded physiological monitoring is an important direction for future work. Course profiles were derived from single-device barometric recordings, which may introduce measurement uncertainty in complex terrain [11,17]. The altitude–VO_2_max correction polynomial approximates the relationship established in endurance athletes tested at real-world altitudes [10]; however, in trail running, the physiological impact of altitude interacts with variable running speed, terrain complexity, and event duration in ways that a single scalar correction does not fully represent. Individual sensitivity to altitude, which varies considerably between athletes of similar aerobic fitness, is not accounted for in the current implementation. Future research should consider this. Finally, the model does not incorporate nutritional intake, hydration status, or equipment-related factors, which may become critical determinants of performance in longer-duration events.

The physics-based architecture of the present model offers a structural advantage over data-driven and machine learning alternatives: the prediction engine requires no historical race data, making individual calibration feasible from as few as 13 observations, a dataset size at which empirical models would be unreliable, and machine learning approaches wholly infeasible. This staged validation philosophy, progressing from mechanistic proof-of-concept through sequential component testing to individual calibration, is consistent with modular implementation frameworks proposed for the development and validation of computational models in sports science [19]. Building on this foundation, two research priorities are proposed. First, a cohort study (n = 15–20 athletes, 5–10 races each) should examine inter-individual variability in α and μ and determine whether these parameters can be reliably estimated from race data alone, without laboratory physiological testing, making the full framework accessible to athletes who lack access to laboratory assessment. Second, a larger cohort (N ≥ 20) incorporating races across a wider range of altitudes and thermal conditions would enable individual calibration of environmental sensitivity coefficients, extending the model from population-level to athlete-specific altitude and heat corrections, the components most likely to limit predictive accuracy in high-mountain or ultra-distance running events. Finally, beyond race prediction, μ may serve as a longitudinal biomarker of fatigue resistance, particularly for trail running: tracking its evolution across a season, without requiring laboratory reassessment, would provide athletes and coaches with a race-derived, ecologically valid index of improvements in aerobic durability, a construct increasingly recognised as a key determinant of endurance performance [20].

## 5. Conclusions

This proof-of-concept study demonstrates that a physics-based digital twin integrating terrain-dependent biomechanics, barometric course profiling, and individual calibration can accurately predict trail running performance within a single highly trained athlete across heterogeneous race conditions. Terrain-driven locomotion cost emerged as the primary determinant of performance variability, with slope-dependent corrections providing the largest improvement in predictive accuracy, highlighting the limitations of flat-road models in heterogeneous environments. The framework enables segment-by-segment simulation of race performance while retaining physiological interpretability through athlete-specific parameters. However, prediction errors in technically demanding races indicate that terrain technicality remains an important unmodelled factor. Future work should extend validation across diverse athlete populations and incorporate surface-related biomechanical constraints to further improve model accuracy and ecological validity.

## Figures and Tables

**Table 1:** Race characteristics and in-sample prediction results across 13 trail-running races. Values include race distance, cumulative positive elevation gain (D+), actual and predicted finish times under the calibrated model (α = 0.92, μ = 0.00), and relative prediction error. Error (%) was calculated as (predicted − actual)/actual × 100. Mean ± SD values are reported for descriptive variables.

Race	Date	Distance (km)	D+ (m)	Actual Time (min)	Predicted Time (min)	Error (%)
1	5 April 2025	28.3	2037	167.5	155.3	−7.3
2	19 April 2025	20.9	1870	169.5	150.4	−11.3
3	26 April 2025	23.3	1445	146.1	122.1	−16.4
4	25 May 2025	41.5	2552	234.3	222.9	−4.9
5	8 June 2025	33.6	1658	176.4	163.4	−7.4
6	6 September 2025	11.5	1763	83.9	108.6	+29.5
7	26 September 2025	43.2	3429	315.0	266.4	−15.4
8	12 October 2025	20.4	1721	134.6	120.3	−10.6
9	9 November 2025	53.2	914	223.9	221.2	−1.2
10	22 February 2026	46.2	2491	240.6	229.7	−4.5
11	22 March 2026	46.1	1977	191.5	214.1	+11.8
12	11 April 2026	29.2	2166	160.3	160.9	+0.4
13	1 May 2026	23.2	1475	111.7	120.8	+8.1
Mean ± SD		32.4 ± 12.6	1961 ± 621	181.2 ± 61.0	173.5 ± 51.4	−2.2 ± 12.6

## Data Availability

Data may be obtained from the corresponding author based upon a reasonable request.

## Abbreviations

The following abbreviations are used in this manuscript:

BMI	Body mass index
CTL	Chronic training load
CV	Cross-validation
ERA5	ECMWF Reanalysis v5
GAP	Grade-adjusted pace
HR	Heart rate
IMUs	Inertial measurement units
LOO-CV	Leave-one-out cross-validation
CLRO	Level-running cost
MAE	Mean absolute error
MAPE	Mean absolute percentage error
R^2^	Coefficient of determination
SD	Standard deviation
TRIMP	Training Impulse
TSB	Training stress balance
VO_2_max	Maximal oxygen uptake
VT_2_	Second ventilatory threshold
WBGT	Wet-bulb globe temperature
